# Association between healthy lifestyle and the risk of ischemic stroke among elderly adults with hypertension: a cross-sectional study in China

**DOI:** 10.3389/fnut.2025.1677786

**Published:** 2025-11-19

**Authors:** Jian Wu, Xiaoyu Jiao, Wenzhe Qi, Lipei Zhao, Xinghong Guo, Nengguang Dai, Rongmei Liu, Qiuping Zhao, Yudong Miao, Clifford Silver Tarimo, Beizhu Ye

**Affiliations:** 1 Department of Health Management, College of Public Health, Zhengzhou University, Zhengzhou, Henan, China; 2 Grassroots Health and Health Department, Health Commission of Henan Province, Zhengzhou, Henan, China; 3 Henan Key Laboratory for Health Management of Chronic Diseases, Central China Fuwai Hospital, Central China Fuwai Hospital of Zhengzhou University, Zhengzhou, Henan, China; 4 Department of Science and Laboratory Technology, Dar es salaam Institute of Technology, Dar es Salaam, Tanzania

**Keywords:** elderly, ischemic stroke, hypertensive patients, healthy lifestyle, GMDR

## Abstract

**Introduction:**

While adopting multiple healthy lifestyle behaviors has been shown to significantly reduce the risk of ischemic stroke (IS) in the general adult population, there is limited evidence on whether such benefits extend to older adults with hypertension. This study aimed to examine how a combination of modifiable healthy lifestyle behaviors is associated with the risks of IS among hypertensive patients aged 65 years and older.

**Methods:**

This population-based study was conducted in Jia County, Henan Province, from 1 July, 2023 to 31 August, 2023. Data on participants’ lifestyles were collected through structured, face-to-face interviews. A composite lifestyle score was generated using five modifiable behaviors: non-smoking, non-drinking, ideal sleep duration, adherence to a healthy diet, and engagement in regular physical activity. The relationship between lifestyle and IS was determined using logistic regression models, with results presented as odds ratios (ORs) and 95% confidence intervals (CIs). To identify optimal interaction patterns among multiple factors, we applied generalized multifactor dimensionality reduction (GMDR) analysis.

**Results:**

A total of 17,747 participants were included (42.11% male, mean age 73.39 years), 31.20% of whom had a history of IS. In multivariable-adjusted models, maintaining a healthy diet, never smoking, getting adequate sleep, and never drinking were each independently associated with a lower risk of IS. There was a clear inverse relationship between the number of healthy lifestyle behaviors and IS risk. After adjusting for covariates, participants who adopted all five healthy lifestyle behaviors had the lowest prevalence of IS, with a 58.5% reduction compared to those who reported none of the healthy behaviors. For every additional point gained in the healthy lifestyle score, the risk of IS dropped by 11.2%. The GMDR analysis showed that sleep, diet, and smoking had the most significant interaction with the risk of IS.

**Conclusion:**

Embracing healthy lifestyle habits can significantly lower the risk of IS among older adults with hypertension in China. These findings offer valuable guidance for designing targeted lifestyle interventions aimed at preventing stroke in this high-risk population.

## 1 Introduction

The global population is aging at a rapid pace, driven by increaing life expectancy and improved survival among older adults, largely attributable to advances in healthcare and broader socioeconomic development (1). By 2050, an estimated 20% of the global population will be aged 65 years and older, with approximately 80% of older adults residing in low- and middle-income countries, posing substantial public health challenges (2). In China, individuals aged 65 years and older currently account for 15.4% of the total population (3). The rapid aging of the population has been accompanied by a rise in chronic diseases such as hypertension, imposing a significant burden on health systems. Hypertension is a leading cause of cardiovascular disease and premature deaths worldwide, with its global prevalence estimated to exceed 40% (4). In China, an estimated 245 million people are living with hypertension, with its prevalence increasing markedly with age and affecting more than half of those aged 65 years and older (5). Developing effective strategies to address health issues related to population aging has therefore become both urgent and unavoidable.

Ischemic stroke (IS) poses a substantial burden on global health and remains the second leading cause of death worldwide, posing a serious threat to health and quality of life (6). By 2030, the global age-standardized IS incidence rate is projected to rise to 89.32 per 100,000 persons (6). The prevalence of stroke in China is severe and continues to increase. Currently, the average age of stroke patients has reached 65 years, and IS accounts for 81.90% of all stroke events (7). Emerging evidence indicates that older adults with hypertension exhibit a substantially greater risk of cardio-cerebrovascular disease (8). Another study showed that blood pressure was independently associated with IS risk and served as a significant contributing factor for IS recurrence (9). Therefore, it is essential to constantly emphasize the significance of preventing IS among hypertensive patients aged 65 and older.

Evidence from previous studies has identified several factors that may help lower the risk of IS, with growing attention focused on lifestyle choices as key modifiable behaviors in prevention efforts (10, 11). Healthy lifestyles can significantly reduce the risk of IS by strengthening the body’s immunity and achieving optimal metabolic function. Compelling evidence suggested that adhering to healthy lifestyles, such as not smoking, avoiding alcohol, getting adequate sleep, engaging in regular physical activity, and following a healthy diet, can significantly lower the risk of IS (12, 13). This is attributable to the fact that healthy lifestyles can enhance the body’s immunity and optimize metabolic functions, thereby significantly reducing the risk of IS. In addition, up to 90% of stroke cases may be prevented in the early stages, with half of these cases attributable to lifestyle modification. A study found that individuals who followed none or only one healthy lifestyle behavior had a 66% higher risk of stroke compared to those who adopted three or four healthy lifestyle practices (14). Nonetheless, a significant research gap persists concerning elderly patients with hypertension, especially those aged 65 years and above. The effect of both individual and composite lifestyle behaviors in this demographic on the risk of IS has yet to be thoroughly investigated.

Our study aimed to examine the effect of both individual and combined lifestyle behaviors on IS risk in hypertensive adults aged 65 years and older. Additionally, we aimed to identify significant and optimal multifactorial interaction patterns among five key lifestyle factors using generalized multifactor dimensionality reduction (GMDR). The findings of this study are expected to offer valuable insights into the development of targeted lifestyle interventions for older hypertensive individuals, ultimately supporting improved health outcomes and wellbeing in this population.

## 2 Materials and methods

### 2.1 Study design and participants

**Figure 1:**
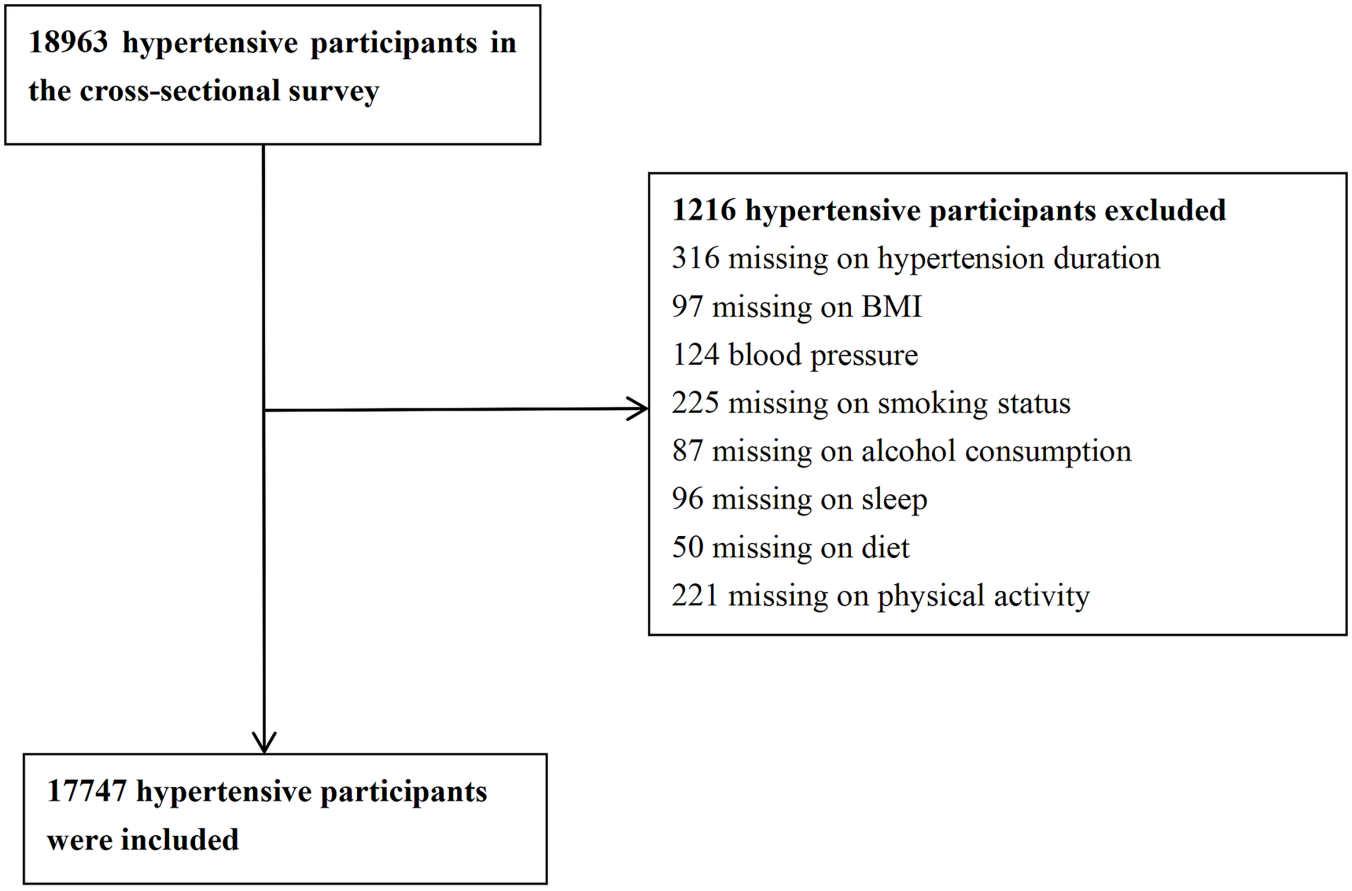
Flowchart of participant recruitment.

We used data from a cross-section survey of 18,963 patients with primary hypertension aged 65 and above in Jia County, Henan Province, from 1 July to 31 August, 2023, selected through a cluster sampling method. All recruited participants were drawn from the National Basic Public Health Service Project (NBPHSP) and had received a confirmed diagnosis from qualified medical professionals. Questionnaire information was obtained through face-to-face interviews. Participants were included in the study if they provided informed consent, completed the questionnaire, and underwent a physical examination conducted by professionally trained investigators. We excluded participants with missing information on healthy lifestyle behaviors (*n* = 679), missing physical examination data (*n* = 413), or missing information on the duration of hypertension (*n* = 316). After these exclusions, 17,747 participants remained for analysis. The flowchart of participant selection is shown in Figure 1. Investigators received training in face-to-face interviewing, physical measurement tools, and questionnaire quality control and passed the examination. The study was approved by the Zhengzhou University Medical Ethics Committee (Approval number: 2023-318).

### 2.2 Assessment of lifestyle behaviors

We assessed five modifiable lifestyle behaviors, including smoking, drinking, sleep, diet, and physical activity. As for smoking, participants were categorized as current smokers and never smokers, with never smokers defined as those who had never smoked or had quit smoking for at least 30 years (15). Never smoking was defined as a healthy lifestyle. Participants’ alcohol consumption was categorized as current drinkers or never drinkers, with never drinking defined as having a healthy lifestyle (16). Dietary information was collected by recording how frequently participants consumed three key food items: fruits, vegetables, and red meat. A healthy diet was defined as meeting the following three criteria: fruits: ≥7times/week; vegetables: ≥7times/week; red meats: ≤6 times/week. Sleep information was based on participants’ reported actual sleep duration per night. An ideal sleep duration, defined as 6 to 8 h per night, was considered a healthy lifestyle behavior, in line with cutoffs commonly used in previous studies to ensure comparability (17). For physical activity, data on the weekly frequency and duration of exercise were collected. Regular exercise was defined as engaging in at least 3.5 h/week of moderate to vigorous–intensity exercise (≥30 min/day) as a healthy lifestyle, in accordance with the WHO guidelines.

We evaluated the association of each single lifestyle behavior with the risk of IS. For further investigation of the combined effect of lifestyle behaviors on the risk of IS, the participant received a score of 1 if they met the criterion for healthy lifestyle behavior and a score of 0 if otherwise. The scores on all lifestyle components were summed to yield a healthy lifestyle score ranging from 0 to 5, with a higher index indicating a healthier lifestyle.

### 2.3 Outcome

In this study, the prevalence of IS in this population was obtained from the NBPHSP. The classification of diseases was adopted in the International Classification of Diseases (ICD-10), and IS [codes I63] was used as the outcome variable in this study to investigate whether and how lifestyle influenced the risk of IS in hypertensive patients.

### 2.4 Covariates

Based on the existing literature, we identified a series of covariates. Sociodemographic information included age based on median (≤72 years, >72 years), sex (male or female), ethnicity (Han or Hui), marital status (married, widowed, or divorced/single/other status), years of education (≤6 year, 7–9years, ≥10years), and yearly household income (≤CNY¥30,000, >CNY¥30,000).

Health-related covariates were also collected, including hypertension duration, body mass index (BMI), and blood pressure. All participants’ height and weight were measured twice following a standard protocol, and the mean was used for analysis. BMI was weight divided by height squared. According to Chinese BMI classification standards, a normal body mass index (BMI) was defined as ranging from 18.5 to 23.99 kg/m^2^, while values outside this range were considered abnormal (18). Blood pressure was measured after the participant sat still for 5 min. The measurement of blood pressure was repeated three times, and the average value was adopted as the blood pressure. In accordance with the Chinese guidelines for the management of hypertension, hypertension was defined as having a systolic blood pressure (SBP) ≥140 mmHg (18.6 kPa) and/or a diastolic blood pressure (DBP) ≥90 mmHg (12.0 kPa) in the absence of antihypertensive medication, or having a documented history of hypertension, or currently taking antihypertensive drugs (19). All measurements were carried out by trained staff using standard instruments.

### 2.5 Statistical analysis

Characteristics of participants were described as counts (percentages) for categorical variables. Missing values are summarized in Supplementary Table S1. A multiple imputation chain-equation was applied to address missing demographic data. The chi-squared test was used to compare the differences in categorical variables between the IS group and non-ischemic stroke group. A binary logistic regression model was deployed to examine the associations between the prevalence of IS and each sociodemographic factor, lifestyle behaviors, and the healthy lifestyle score. Three models were used to examine the associations between healthy lifestyle scores and the prevalence of IS. Model 1 included no covariate adjustments. Model 2 was adjusted for sociodemographic factors, including age, sex, ethnicity, marital status, years of education, and household income. Model 3 further accounted for clinical variables such as the duration of hypertension, body mass index (BMI), and blood pressure. We implemented the trend test by adopting the categorical variable of a healthy lifestyle as a continuous variable. Using GMDR, the study identified statistically significant and optimal interaction models among the five healthy lifestyle behaviors. Parameters consisted of the sign test *p*-value, cross-validation consistency (CVC), and test balance accuracy (TBA). The optimal model was determined to be the one with the highest TBA, a low symbolic test *p*-value (≤0.05), and the highest CVC score.

A subgroup analysis based on participants’ age was conducted to explore the differences in the effect of lifestyle scores on health outcomes in different subgroups. In each subgroup, the reference group was set as the participants with the least healthy lifestyle behavior. To check the robustness of the results, we performed two sensitivity analyses. First, we redefined physical exercise; at least 150 min of moderate or 75 min of vigorous activity per week was considered a healthy behavior according to American adult physical activity guidelines (20). Second, as some studies treat BMI as a lifestyle factor (21), we incorporated BMI into the lifestyle score to re-evaluate the association between combined lifestyle factors and the risk of IS. All statistical analyses were conducted with SPSS software (version 26), R software (version 4.3.3), and GMDR (version 1.0). A two-sided *p*-value of < 0.05 was considered statistically significant.

## 3 Results

### 3.1 Characteristics of study participants

A total of 17,747 participants were screened for eligibility (Figure 1). Table 1 presents the participants’ demographic characteristics stratified by IS status. The median age of the participants was 72 years, and 42.11% were male. Among all participants with hypertension, 5,537 (31.20%) of them had a history of IS. The prevalence of IS was significantly higher among men (32.85%), individuals with lower income (31.47%), participants with >5 years of hypertension (32.90%), and participants with an abnormal BMI (31.77%) (*p* < 0.001). No statistically significant differences were observed with respect to age, marital status, or blood pressure (*p* > 0.05).

**Table 1:** Characteristics of study participants.

Characteristic	Total (*N* = 17,747)	Ischemic stroke (*N* = 5,537)	No ischemic stroke(N = 12,210)	*χ* ^2^	*p*-value
Age, years (%)				1.208	0.278
≤72	8846 (49.85)	2726 (30.82)	6120 (69.18)		
>72	8901 (50.15)	2811 (31.58)	6090 (68.42)		
Sex(%)				16.553	<0.001
Male	7473 (42.11)	2455 (32.85)	5018 (67.15)		
Female	10274 (57.89)	3082 (30.00)	7192 (70.00)		
Ethnicity				12.391	<0.001
Han nationality	17541 (98.84)	5496 (31.33)	12045 (68.67)		
Hui nationality	206 (1.16)	41 (19.90)	165 (80.10)		
Marital status (%)				0.688	0.709
Married	12706 (71.59)	3987 (31.38)	8719 (68.62)		
Widowed	4353 (24.53)	1340 (30.78)	3013 (69.22)		
Divorced, single, and other status	688 (3.88)	210 (30.52)	478(69.48)		
Years of education (%)				10.216	0.006
≤6	13885 (78.24)	4373 (31.49)	9512 (68.51)		
7–9	2933 (16.53)	918 (31.30)	2015 (68.70)		
≥10	929 (5.23)	246 (26.48)	683 (73.52)		
Yearly household income (Chinese Yuan)(%)				7.127	0.008
≤30,000	16355 (92.16)	5147 (31.47)	11208 (68.53)		
>30,000	1392 (7.84)	390 (28.02)	1002 (71.98)		
Hypertension duration, years (%)				204.843	<0.001
≤5	5673 (31.97)	1358 (24.09)	4315 (75.91)		
>5	12074 (68.03)	4179 (32.90)	7895 (67.10)		
BMI (%)				4.101	0.043
Abnormal	10708 (60.34)	3402 (31.77)	7306 (68.23)		
Normal	7039 (39.66)	2135 (30.33)	4904 (69.67)		
Blood pressure (%)				0.776	0.380
Normal	8128 (45.80)	2563 (31.53)	5565 (68.47)		
High	9619 (54.20)	2974 (30.92)	6645 (69.08)		
Smoking status (%)				41.799	<0.001
Current	4243 (23.91)	1494 (35.21)	2749 (64.79)		
Never	13504 (76.09)	4043 (29.94)	9461 (70.06)		
Drinking status (%)				6.806	0.010
Current	2056 (11.59)	693 (33.71)	1363 (66.29)		
Never	15691 (88.41)	4844 (30.87)	10847 (69.13)		
Sleep duration (%)				39.913	<0.001
Non-ideal	6809 (38.37)	2314 (33.98)	4495 (66.02)		
Ideal	10938 (61.63)	3223 (29.47)	7715 (70.53)		
Healthy Diet (%)				27.826	<0.001
No	16049 (90.43)	5013 (31.24)	10946 (68.76)		
Yes	1698 (9.57)	434 (25.56)	1264 (74.43)		
Physical activity (%)				2.606	0.107
Irregular	8018 (45.18)	2452 (30.58)	5566 (69.42)		
Regular	9729 (54.82)	3085 (31.71)	6644 (68.29)		
Healthy lifestyle score (%)				52.633	<0.001
0	242 (1.36)	110 (45.45)	132 (54.55)		
1	1284 (7.24)	464 (36.14)	820 (63.86)		
2	4030 (22.70)	1325 (32.88)	2705 (67.12)		
3	7044 (39.69)	2162 (30.69)	1882 (69.31)		
4	4651 (26.21)	1351 (29.04)	3300 (70.96)		
5	496 (2.80)	125 (25.20)	371 (74.80)		

BMI, body mass index.

A total of 13,504 (76.09%) participants reported never smoking, 15,691 (88.41%) participants reported never drinking, 10,938 (61.63%) participants reported ideal sleep duration, 1,698 (9.57%) participants reported a healthy diet, and 9,729 (54.82%) participants reported regular physical activity. The proportions of participants with a healthy lifestyle score of 0, 1, 2, 3, 4, and 5 were 242 (1.36%), 1,284 (7.24%), 4,030 (22.70%), 7,044 (39.69%), 4,651 (26.21%), and 496 (2.80%), respectively.

### 3.2 Associations of demographic information with IS

**Figure 2:**
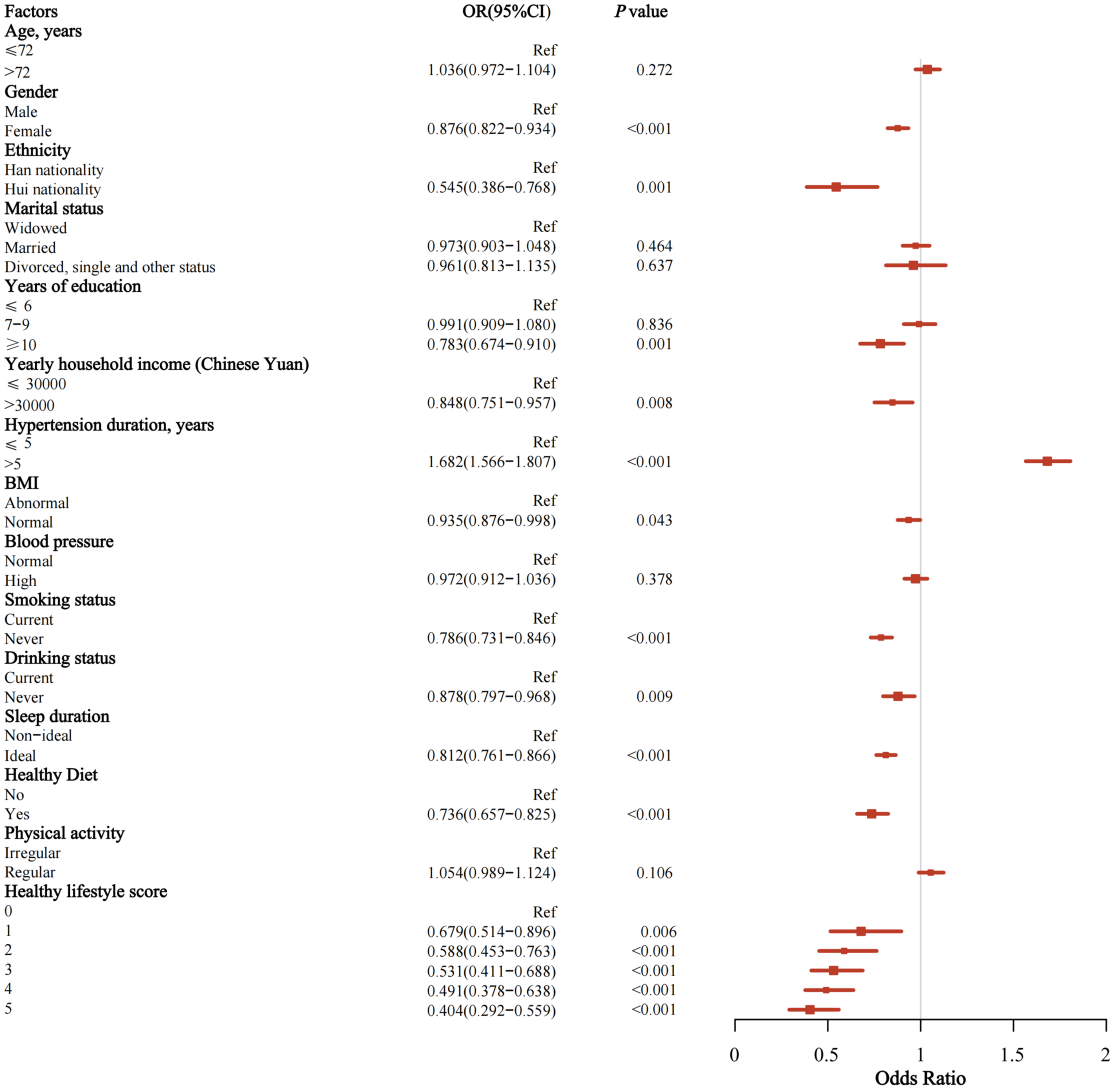
Forest map of the multivariate logistic regression analysis.

The multivariate regression analysis indicated that being female (OR = 0.876; 95% CI = 0.822–0.934), belonging to the Hui ethnic group (OR = 0.545; 95% CI = 0.386–0.768), having a higher income level (annual income >30,000) (OR = 0.848; 95% CI = 0.751–0.957), and having a normal BMI (OR = 0.935; 95% CI = 0.876–0.998) were associated with a protective effect against IS. Conversely, a hypertension duration of more than 5 years (OR = 1.682; 95% CI = 1.566–1.807) was associated with an increased risk of IS. Additionally, educational attainment appeared to have a potential impact on the risk of IS (Figure 2).

### 3.3 Associations of individual healthy lifestyle behaviors with the risk of IS

The findings indicated that, among lifestyle factors, maintaining a healthy diet had the strongest protective effect against IS (OR = 0.736; 95% CI = 0.657–0.825), followed by never smoking (OR = 0.786; 95% CI = 0.731–0.846), achieving an ideal sleep duration (OR = 0.812; 95% CI = 0.761–0.866), and never drinking (OR = 0.878; 95% CI = 0.797–0.968). A healthy diet, compared with insufficient intake of fruits, vegetables, and meat; never smoking, compared with current smoking; ideal sleep duration, compared with sleeping more than 8 h or less than 6 h; and never drinking, compared with current drinking, emerged as protective factors against the risk of IS in hypertensive patients (Figure 2; Supplementary Table S2).

### 3.4 Associations of the healthy lifestyle score with IS

In the analysis where the five healthy lifestyle behaviors were considered jointly using a healthy lifestyle score, a higher score was significantly associated with a lower risk of IS, showing a non-linear dose-response relationship after adjusting for confounders (all *p* < 0.05, Table 2). After adjusting for the potential confounding factors in model 2 (age, gender, nation, marital status, education, and household income), the prevalence of IS was found to be lowest when the score of healthy lifestyle behaviors reached 5, with a decrease of 57.9% (OR = 0.85; 95% CI = 0.303–0.584) compared to a healthy lifestyle behavior score of 0. In model 3, which additionally controlled for hypertension duration, BMI, and blood pressure, the results remained robust. Compared to participants who had a healthy lifestyle behavior score of 0, the risk of IS decreased by 32.3% (OR = 0.677; 95% CI = 0.512–0.896) among participants with a healthy lifestyle behavior scored of 1, 40.1% (OR = 0.599; 95% CI = 0.459–0.781) among participants who had a healthy lifestyle behavior scored of 2, 45.7% (OR = 0.543; 95% CI = 0.417–0.708) among participants who had a healthy lifestyle behavior scored of 3, 49.7% (OR = 0.503; 95% CI = 0.386–0.661) among participants who had a healthy lifestyle behavior scored of 4, and 58.5% (OR = 0.415; 95% CI = 0.298–0.579) among participants who had a healthy lifestyle behavior scored of 5 (Table 2).

**Table 2:** Association between healthy lifestyles and risk of stroke.

Healthy lifestyle score	Model 1			Model 2			Model 3		
	OR (95% CI)	*p*-value	*p-value* for trend	OR (95% CI)	*p*-value	*P-value* for trend	OR (95% CI)	*p*-value	*p*-value for trend
0	Ref		<0.001	Ref		<0.001	Ref		<0.001
1	0.679 (0.514–0.896)	0.006		0.677 (0.513–0.894)	0.006		0.677 (0.512–0.896)	0.006	
2	0.588 (0.453–0.763)	<0.001		0.596 (0.458–0.776)	<0.001		0.599 (0.459–0.781)	<0.001	
3	0.531 (0.411–0.688)	<0.001		0.545 (0.419–0.709)	<0.001		0.543 (0.417–0.708)	<0.001	
4	0.491 (0.378–0.638)	<0.001		0.505 (0.387–0.660)	<0.001		0.503 (0.386–0.661)	<0.001	
5	0.404 (0.292–0.559)	<0.001		0.421 (0.303–0.584)	<0.001		0.415 (0.298–0.579)	<0.001	

Model 1 did not control for any covariates; model 2 adjusted for age, sex, nationality, marital status, years of education, and income; and model 3 further adjusted for hypertension duration, BMI, and blood pressure. *p* for trend analysis used indicator levels as a continuous variable.

Furthermore, each point gained in the healthy lifestyle score was accompanied by an 11.2% reduction in the risk of IS in hypertensive patients (OR = 0.888; 95% CI = 0.860–0.916). As shown in Supplementary Table S2, a higher healthy lifestyle score was inversely associated with the risk of stroke.

### 3.5 High-Order Interaction

The GMDR analysis was conducted to explore potential interactions among the five healthy lifestyle factors in this study. After constructing higher-order interaction models, the three-factor model, including sleep, diet, and smoking, showed a significant replacement test (*p* < 0.05), and a CVC of 10/10, with the highest balanced accuracy of 0.5350, making it the optimal model (Table 3; Supplementary Figure S1). These results suggest that sleep, diet, and smoking have the strongest interaction effect on the risk of cerebral infarction.

**Table 3:** High-order interaction GMDR model for a healthy lifestyle.

Model	TBA	CVC	*p*-value
Sleep	0.5212	9/10	0.001
Sleep, smoke	0.5310	10/10	0.010
Sleep, diet, smoke	0.5350	10/10	0.001
Sleep, diet, smoke, drink	0.5337	9/10	0.010
Sleep, physical activity, diet, smoke, drink	0.5311	10/10	0.010

GMDR, generalized multifactor dimensionality reduction; TBA, test balance accuracy, and CVC: cross-validation consistency.

### 3.6 Subgroup and sensitivity analyses

Supplementary Table S3 shows stratification by age group. The inverse associations between multiple healthy lifestyle behaviors and the risk of IS remained significant among both age groups. Two sensitivity analyses were conducted to examine the relationship between the healthy lifestyle score and the risk of IS in hypertensive patients. The results indicated that the findings from each sensitivity analysis were basically consistent with the primary analysis results (Supplementary Tables S4, S5).

## 4 Discussion

In our study, we observed that approximately 31.20% of the 17,747 participants aged 65 years and above experienced IS. Our findings revealed that adhering to healthy lifestyles (never smoking, never drinking, ideal sleep duration, and a healthy diet) was associated with a significantly reduced risk of IS, and a healthy diet had the greatest effect on reducing the risk of IS. The prevalence of IS decreased by 58.5% among individuals with a healthy lifestyle behavior score of 5 compared to those with a score of 0. In addition, each additional lifestyle factor was associated with an 11.2% lower prevalence of IS.

Our findings are consistent with previous studies, showing that the risk of IS decreases with increasing scores of healthy lifestyle behaviors (12, 22). A representative population-based prospective cohort study of 60-year-olds showed a 61% lower risk of cardiovascular disease among those with 6–7 healthy behaviors than among those with 0–2 healthy lifestyles (22). Similarly, another prospective cohort study revealed that adopting a healthier lifestyle was associated with a reduced risk of developing IS (23). The results of our study further support the importance of continuous exploration and intensive healthy lifestyle management, with a view to guiding the health management of this population more accurately, reducing the development of cerebral infarction events, and improving overall population health.

In the present study, adhering to the recommendations of ideal sleep duration was shown to have a protective effect on IS in our population. Findings from meta-analyses have shown a statistically significant linear association between short (less than 6 h) and long (more than 9 h) sleep duration and cardiovascular disease and mortality (24, 25). We also observed a higher prevalence of IS among individuals who smoked or consumed alcohol compared to those who had never smoked or never drank. Previous studies have found that smoking and drinking increased the risk of IS, and lifestyle modifications have the potential to reduce IS risk (26, 27), which is in line with our study.

In our study, only 1,698 (9.6%) participants adhered to a healthy diet, which is quite low and consistent with previous studies (28). Previous multiple studies have confirmed that dietary patterns are closely associated with the risk of developing IS. Specifically, high consumption of processed meats, unprocessed red meats, or poultry was significantly related to an increased risk of IS (29). Moreover, a study involving 23,797 participants clearly demonstrated that a high-quality dietary pattern characterized by high intake of fruits and vegetables and low intake of red meat and processed meats was associated with a lower risk of IS (30). Basic medical research suggested that vegetables and fruits are rich in dietary fiber, phytochemicals, vitamins, and other substances. These components can not only inhibit the oxidation of low-density lipoprotein (LDL), thereby reducing atherosclerotic plaques, but also reduce the release of inflammatory factors, inhibit platelet aggregation, prevent thrombosis, and thus slow down the occurrence and development of cerebral infarction (31–33). The results of this study suggested that we should pay close attention to the popularization of dietary knowledge in this group and further optimize the dietary guidelines according to their nutritional needs and dietary characteristics so as to improve their dietary health level and reduce the risk of IS.

However, no significant association was observed between regular physical activity and the risk of IS in this study. The relationship between the intensity of physical activity and the risk of cardio-cerebrovascular disease was controversial in the previous study. A longitudinal study involving 49,060 adults indicated that 150–300 min/week of moderate-intensity physical activity or more was significantly associated with decreased risk of stroke; however, 75–150 min/week of vigorous-intensity physical activity was found to be associated with few further benefits, and it may even weaken the cardiovascular benefits (34). Prestroke physical activity was associated with milder stroke; both light physical activity (such as walking for at least 4 h/week) and moderate physical activity (2–3 h/week) appeared to be beneficial(35). Moreover, another study reported no association between self-reported moderate-to-vigorous physical activity, accelerometer-based physical activity, sedentary behavior, and IS (36). This discrepancy may be due to the insufficient consideration of other potential confounding factors, such as hereditary factors or comorbidity, which can significantly influence IS (16). Additionally, short-term changes in physical activity may not be sufficient to have a significant effect on IS.

Previous GMDR studies focused on the effects of gene-gene and gene-environment interactions on disease (37). To the best of our knowledge, this is the first study to use GMDR to explore possible higher-order interactions between different lifestyle factors and found that the three factors of sleep, diet, and smoking interacted with each other and had the strongest interactions. Our analysis identified a significant three-way interaction involving sleep, diet, and smoking, which exhibited the strongest effect among the models tested. Methodologically, the GMDR approach, utilizing cross-validation and permutation testing, strengthens the reliability of these findings by mitigating the risk of overfitting, a common limitation in traditional additive or multiplicative interaction models (38). These findings deepen our understanding of the complex relationship between health behaviors and IS risk. The significant interplay observed among these modifiable factors underscores that public health interventions must evolve from promoting isolated behavioral changes toward implementing integrated, multi-dimensional strategies. For instance, simultaneously addressing sleep quality, dietary habits, and smoking cessation may yield synergistic effects, amplifying the benefits of primary prevention beyond what could be achieved by targeting each factor individually. Furthermore, our results aid in identifying population subgroups with specific risk profiles, enabling the design of targeted and personalized preventive measures. This approach is anticipated to enhance the precision, efficiency, and overall effectiveness of resource allocation in health programs targeting aging populations with hypertension.

To our knowledge, this is the first study to comprehensively examine health lifestyle behaviors in relation to the prevalence of IS among hypertensive participants aged 65 years and above, emphasizing the potential role of healthy lifestyles in IS.

Moreover, owing to the large, population-based sample, the findings are likely to be generalizable to individuals with primary hypertension residing in low-and middle-income countries. This study also had several limitations. First, the cross-sectional design of the study restricted the exploration of changes in lifestyles or the determination of causality, despite providing insights into the prevalence of IS among older hypertensive patients and its correlation with lifestyle behaviors. Second, the reliance on self-reported information about lifestyle behaviors may introduce information bias. The accuracy of responses from elderly participants with hypertension might be affected by memory decline, despite efforts to mitigate this through questionnaire design and interviewer assistance. Third, despite adjusting for recognized potential factors, the possibility of unmeasured confounders and reverse causation cannot be excluded.

## 5 Conclusion

In conclusion, our findings suggest that a higher healthy lifestyle score, including never smoking, never drinking, ideal sleep duration, a healthy diet, and regular physical activity, is associated with a substantially lower risk of IS among hypertensive patients aged 65 years and above. In particular, sleep, diet, and smoking emerged as the most significant interactive influences on IS risk. These insights provide important evidence to inform the development of tailored guidelines and public health interventions aimed at lowering stroke risk through lifestyle modification in this high-risk demographic.

## Data Availability

The raw data supporting the conclusions of this article will be made available by the authors, without undue reservation.
