# Supplementary material for: Association between healthy lifestyle and the risk of ischemic stroke among elderly adults with hypertension: a cross-sectional study in China
Source: Front Nutr. 2025 Nov 19;12:1677786. doi: 10.3389/fnut.2025.1677786 (PMC12672219; doi:10.3389/fnut.2025.1677786)
Supplement: Supplementary file 1 [file Table_1.docx]

**Supplementary materials**

**Supplementary table 1.** Distribution of missing values.

**Supplementary table 2.** Logistic regression analysis of ischemic stroke-related lifestyles.

**Supplementary table 3.** Risk of ischemic stroke according to lifestyle score stratified by age.

**Supplementary table 4.** The association between lifestyle score and ischemic stroke when using another cut-off values to define physical activity.

**Supplementary table 5.** The association between lifestyle score and ischemic stroke when incorporating BMI into lifestyle.

**Supplementary figure 1.** GMDR model of the interaction of sleep, diet, and smoking with the risk of IS.

**Supplementary table 1. Distribution of missing values**

| **Characteristic** | **Missing value** | **(%)** |
| --- | --- | --- |
| Age | 76 | 0.40 |
| Gender | 4 | 0.02 |
| Nation | 359 | 1.89 |
| Marital status | 47 | 0.25 |
| Years of education | 119 | 0.63 |
| Yearly household income | 1064 | 5.61 |
| Hypertension duration | 316 | 1.67 |
| BMI | 97 | 0.51 |
| Blood pressure | 124 | 0.67 |
| Smoking status | 225 | 1.19 |
| Drinking status | 87 | 0.46 |
| Sleep duration | 96 | 0.51 |
| Healthy Diet | 20 | 0.11 |
| Physical activity | 221 | 1.17 |

BMI – body mass index

**Supplementary table 2. Logistic regression analysis of ischemic stroke-related lifestyles**

| **Characteristic** | **B** | **SE** | **Wals** | ***P*** | **Odd Ratios (95%CI)** |
| --- | --- | --- | --- | --- | --- |
| **Age, years** |  |  |  |  |  |
| ≤72 | ref |  |  |  | Ref |
| ＞72 | 0.036 | 0.032 | 1.208 | 0.272 | 1.036(0.972-1.104) |
| **Gender** |  |  |  |  |  |
| Male | ref |  |  |  | Ref |
| Female | -0.132 | 0.033 | 16.402 | <0.001 | 0.876(0.822-0.934) |
| **Ethnicity** |  |  |  |  |  |
| Han nationality | ref |  |  |  | Ref |
| Hui nationality | -0.608 | 0.175 | 12.025 | 0.001 | 0.545(0.386-0.768) |
| **Marital status** |  |  |  |  |  |
| Widowed | ref |  |  |  | Ref |
| Married | -0.028 | 0.038 | 0.535 | 0.464 | 0.973(0.903-1.048) |
| Divorced, single and other status | -0.040 | 0.085 | 0.222 | 0.637 | 0.961(0.813-1.135) |
| **Years of education** |  |  |  |  |  |
| ≤6 | ref |  |  |  | Ref |
| 7-9 | -0.009 | 0.044 | 0.043 | 0.836 | 0.991(0.909-1.080) |
| ≥ 10 | -0.244 | 0.077 | 10.159 | 0.001 | 0.783(0.674-0.910) |
| **Yearly household income (Chinese Yuan)** |  |  |  |  |  |
| ≤30000 | ref |  |  |  | Ref |
| ＞30000 | -0.165 | 0.062 | 7.113 | 0.008 | 0.848(.751-0.957) |
| **Hypertension duration, years** |  |  |  |  |  |
| ≤5 | ref |  |  |  | Ref |
| ＞5 | 0.520 | 0.037 | 202.629 | <0.001 | 1.682(1.566-1.807) |
| **BMI** |  |  |  |  |  |
| Abnormal | ref |  |  |  | Ref |
| Normal | -0.167 | 0.033 | 4.100 | 0.043 | 0.935(0.876-0.998) |
| **Blood pressure** |  |  |  |  |  |
| Normal | ref |  |  |  | Ref |
| High | -0.029 | 0.033 | 0.776 | 0.378 | 0.972(0.912-1.036) |
| **Smoking status** |  |  |  |  |  |
| Current | ref |  |  |  | Ref |
| Never | -0.240 | 0.037 | 41.696 | <0.001 | 0.786(0.731-0.846) |
| **Drinking status** |  |  |  |  |  |
| Current | ref |  |  |  | Ref |
| Never | -0.130 | 0.050 | 6.800 | 0.009 | 0.878(0.797-0.968) |
| **Sleep duration** |  |  |  |  |  |
| Non-ideal | ref |  |  |  | Ref |
| Ideal | -0.209 | 0.033 | 39.858 | <0.001 | 0.812(0.761-0.866) |
| **Healthy Diet** |  |  |  |  |  |
| Yes | ref |  |  |  | Ref |
| No | -0.306 | 0.058 | 27.654 | <0.001 | 0.736(0.657-0.825) |
| **Physical activity** |  |  |  |  |  |
| Irregular | ref |  |  |  | Ref |
| Regular | 0.053 | 0.033 | 2.606 | 0.106 | 1.054(0.989-1.124) |
| **Healthy lifestyle score** |  |  |  |  |  |
| 0 | ref |  |  |  | Ref |
| 1 | -0.387 | 0.142 | 7.477 | 0.006 | 0.679(0.514-0.896) |
| 2 | -0.531 | 0.133 | 15.870 | <0.001 | 0.588(0.453-0.763) |
| 3 | -0.632 | 0.132 | 23.057 | <0.001 | 0.531(0.411-0.688) |
| 4 | -0.711 | 0.133 | 28.525 | <0.001 | 0.491(0.378-0.638) |
| 5 | -0.906 | 0.165 | 29.970 | <0.001 | 0.404(0.292-0.559) |
| **Each lifestyle factor** | -0.119 | 0.016 | 54.553 | <0.001 | 0.888(0.860-0.916) |

BMI – body mass index, CI – confidence interval, OR – odds ratio, Ref – reference

**Supplementary table 3. Risk of ischemic stroke according to lifestyle score stratified by age**

| **Healthy lifestyle score** | **Age ≤72 (n=8846)** | | ***P*** value for trend | **Age** >**72(n=8901)** | | ***P*** value for trend |
| --- | --- | --- | --- | --- | --- | --- |
|  | **OR (95% CI)** | ***P* Value** |  | **OR (95% CI)** | ***P* Value** |  |
| 0 | Ref |  | <0.001 | Ref |  | <0.001 |
| 1 | 0.590  (0.405-0.859) | 0.006 |  | 0.792（0.524-1.197） | 0.269 |  |
| 2 | 0.538  (0.378-0.765) | 0.001 |  | 0.647（0.438-0.956） | 0.029 |  |
| 3 | 0.508  (0.359-0.719) | <0.001 |  | 0.560（0.381-0.834） | 0.003 |  |
| 4 | 0.491  (0.346-0.698) | <0.001 |  | 0.495（0.335-0.732） | <0.001 |  |
| 5 | 0.361  (0.230-0.564) | <0.001 |  | 0.456（0.284-0.731） | 0.001 |  |

OR – odds ratio, CI – confidence interval

**Supplementary table 4. The association between lifestyle score and ischemic stroke when using another cut-off values to define physical activity**

| **Healthy lifestyle**  **score** | **Ischemic stroke /**  **Non-Ischemic stroke** | **OR (95% CI)** | ***P* value** | ***P* for trend** |
| --- | --- | --- | --- | --- |
| 0 | 76/68 | Ref |  | <0.001 |
| 1 | 322/554 | 0.852(0.615-1.180) | 0.335 |  |
| 2 | 971/1960 | 0.757(0.554-1.033) | 0.079 |  |
| 3 | 1865/3977 | 0.643(0.472-0.876) | 0.005 |  |
| 4 | 1649/3960 | 0.630(0.461-0.861) | 0.004 |  |
| 5 | 615/1556 | 0.471(0.330-0.673) | <0.001 |  |

OR-odds ratio, CI-confidence interval.

Healthy lifestyle including never smoking, never alcohol drinking, ideal sleep duration, healthy diet and regular physical activity. Model adjusted for age, sex, education levels, income, hypertension duration, BMI and blood pressure, further adjusted for physical activity. At least 50 min of moderate or 75 min of vigorous activity per week was considered a healthy factor.

**Supplementary table 5. The association between lifestyle score and ischemic stroke**

| **Healthy lifestyle score** | **Ischemic stroke /**  **Non-Ischemic stroke** | OR **(95%** CI**)** | P **value** | P **for trend** |
| --- | --- | --- | --- | --- |
| 0 | 76/68 | Ref |  | <0.001 |
| 1 | 322/554 | 0.527(0.368-0.753) | <0.001 |  |
| 2 | 971/1960 | 0.458(0.326-0.643) | <0.001 |  |
| 3 | 1865/3977 | 0.438(0.313-0.614) | <0.001 |  |
| 4 | 1649/3960 | 0.391(0.279-0.549) | <0.001 |  |
| 5 | 615/1556 | 0.376(0.266-0.533) | <0.001 |  |
| 6 | 39/135 | 0.267(0.164-0.436) | <0.001 |  |

OR-odds ratio, CI-confidence interval

Healthy lifestyle including never smoking, never alcohol drinking, ideal sleep duration, healthy diet, regular physical activity and normal BMI. Model adjusted for age, sex, education levels, income, hypertension duration and blood pressure.


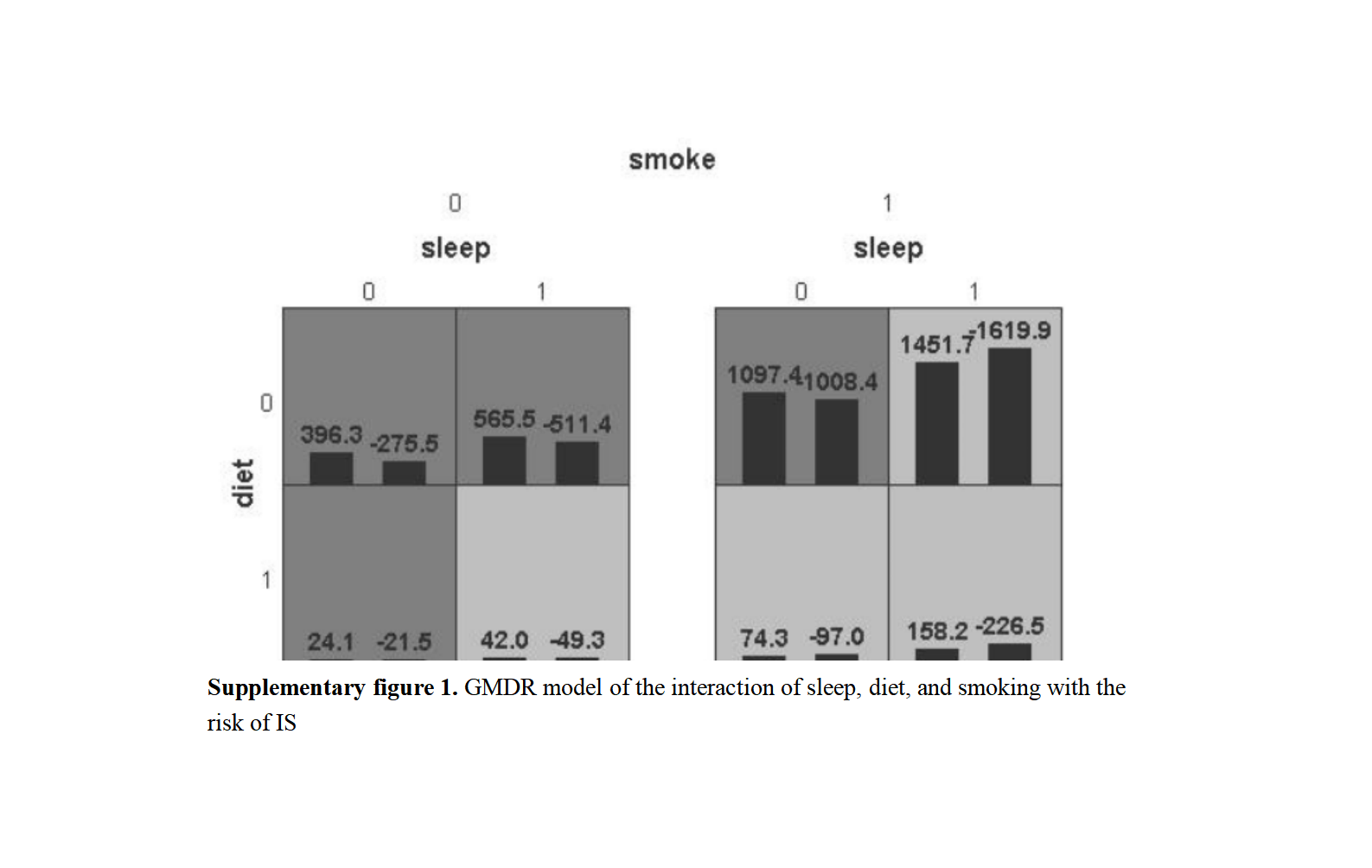


**Supplementary figure 1.** GMDR model of the interaction of sleep, diet, and smoking with the risk of IS
